# Why Are Internal Mammary (Thoracic) Arteries Less Prone to Developing Atherosclerosis Compared to Coronary Arteries? Do Gut Microbiota Play a Role? A Narrative Review

**DOI:** 10.3390/ijms26189052

**Published:** 2025-09-17

**Authors:** Leon M. T. Dicks

**Affiliations:** Department of Microbiology, Stellenbosch University, Private Bag X1, Matieland, Stellenbosch 7602, South Africa; lmtd@sun.ac.za

**Keywords:** atherosclerosis, coronary arteries, internal mammary arteries, gut microbiota

## Abstract

Atherosclerosis (AS), the leading cause of cardiovascular disease (CVD), is the thickening and stiffening of arterial walls, mainly of coronary arteries, the aorta, and the internal carotid artery. Blood flow is restricted by the deposit of lipid-rich macrophages (foam cells), calcium, fibrin, and cellular debris into plaques on the inner lining (tunica intima) of arterial walls. Damaged endothelia become inflamed and accumulate macrophages, monocytes, granulocytes, and dendritic cells, which intensifies plaque formation and increases the risk of myocardial infarction (MI) and thrombosis. Many of the anatomical and physiological abnormalities in arterial walls can be linked to colonic bacteria that produce inflammation-inducing metabolites, e.g., succinate, fumarate, fatty acids (FAs), reactive oxygen species (ROS), lipopolysaccharides (LPS), and trimethylamine-N-oxide (TMAO). TMAO triggers platelet formation, inhibits the synthesis of bile acids (BAs), accelerates the formation of aortic lesions, and upregulates the expression of membrane glycoprotein CD36 (also known as platelet glycoprotein 4) on the surface of platelets and epithelial cells. The ability of internal mammary arteries (IMAs) to produce higher levels of apolipoprotein C-III (apo-CIII) and paraoxonase (PON), compared to coronary arteries, prevents plaque buildup. The tunica intima of IMAs is rich in heparin sulfate and endothelial nitric oxide synthase (eNOS). Increased production of NO relaxes VSMCs and suppresses GTP cyclohydrolase (GTPCH), which lowers blood pressure. Higher levels of prostacyclin (PG12) produced by IMAs inhibit platelet aggregation. IMAs are structurally different from coronary arteries by having a thinner, non-fenestrated, tunica intima without a prominent internal elastic lamina. These characteristics render IMAs ideal conduits in coronary artery bypass graft (CABG) surgery. This review provides information that may explain why IMAs are less affected by inflammatory reactions and more resilient to plaque formation.

## 1. Introduction

**Figure 1:**
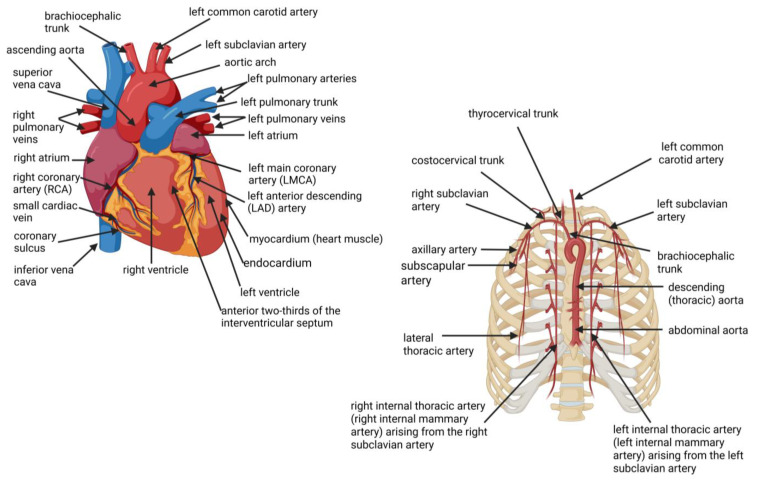
A schematic representation of the heart and main arteries (**left image**) and the internal mammary arteries (IMAs, **right image**). The right coronary artery (RCA, **left image**) emerges from the anterior ascending aorta, supplying blood to the right ventricle, the right atrium, and sinoatrial (SA) and atrioventricular (AV) nodes, regulating the heart rhythm. The RCA divides into the right posterior descending artery (PDA) and the acute marginal artery. The PDA supplies blood to the posterior one-third of the interventricular septum. The left main coronary artery (LMCA) is separated into two branches, the left anterior descending artery (LAD), which supplies blood to the anterior part of the left ventricle and two-thirds of the interventricular septum (**left image**). The myocardium swirls and spirals around the heart chambers in a figure-8 pattern. The internal mammary arteries (IMAs, **right image**), also referred to as the internal thoracic arteries, stem from the proximal area of the brachiocephalic trunk and subclavian artery. The brachiocephalic artery separates into two arteries, the right common carotid artery and the right subclavian artery. The subclavian arteries give rise to the vertebral artery, internal thoracic artery, thyrocervical trunk, costocervical trunk, and dorsal scapular artery. Created using Biorender.com (accessed on 4 September 2025).

Atherosclerosis (AS) is a progressive, lipid-driven inflammatory disease of coronary arteries, the aorta, and the internal carotid artery, leading to plaque formation, fibrosis, the accumulation of macrophage foam cells (Figure 1), and an increase in reactive oxygen species (ROS) [1,2,3]. These arteries share distinct layers: the inner tunica intima, closest to the lumen; the middle tunica media, containing smooth muscle and elastic tissue; and the tissue-rich outer tunica adventitia [4]. The tunica intima in all blood vessels, including large arteries, veins, and capillaries, consists of a single layer of endothelial cells lining the lumen. However, in larger vessels, the tunica intima also contains a subendothelial layer of connective tissue and an internal elastic lamina, which are not present in smaller vessels such as capillaries [4]. Blood flow is restricted by plaques, formed from the accumulation of lipid-rich macrophages (foam cells), calcium, fibrin, and cellular debris deposited on the inner lining (intima) of arterial walls [5,6,7]. Although diet and genetics influence AS, other major risk factors include stress, smoking, abnormal alcohol consumption, lack of exercise, and diabetes [8,9,10,11,12].

Lifestyle has a major effect on the composition of the gut microbiome. Many of the physiological abnormalities are due to inflammatory metabolites produced by gut microbiota. These include succinate, which inhibits prolyl hydroxylase (PHD), an enzyme that usually targets hypoxia-inducible factor-1α (HIF-1α) [13], and fumarate that accumulates in mitochondria and orchestrates the release of mitochondrial DNA and RNA in vesicles [14]. Fatty acids, such as butyrate, and reactive oxygen species (ROS) produced by gut bacteria interact with signaling pathways and cell membrane receptors, e.g., Toll-like receptor 4 (TLR4), platelet glycoprotein 4 (CD36), and G protein receptors (GPRs) [15]. Activation of the TLR4 pathway increases the activity of nicotinamide adenine dinucleotide phosphate (NADPH) oxidase (NOX), nuclear factor kappa B (NF-κB), and mitogen-activated protein kinase (MAPK) pathways. The upregulation of these pathways leads to an increase in pro-inflammatory cytokines [16] and the formation of plaques [17]. An increase in NF-κB activates endothelial nitric oxide synthase (eNOS) and increases nitric oxide (NO) levels. NO relaxes vascular smooth muscle cells (VSMCs) and may ease the restriction of arteries [18,19,20]. Trimethylamine-N-oxide (TMAO) activates Nod-like receptors (NLRs), such as NLR protein 3 (NLRP3), reviewed by Dicks [21], and triggers platelet formation that leads to AS, arterial inflammation, and thrombosis [22,23]. TMAO also inhibits the synthesis of bile acids (BAs), accelerates the formation of aortic lesions by activating Farnesoid receptors (FXRs) [24], and upregulates the expression of CD36 on the surfaces of platelets, mononuclear phagocytes, adipocytes, hepatocytes, myocytes, and epithelial cells [25]. CD36, a scavenger of fatty acids and fatty acid transferase (FAT), also binds to collagen and thrombospondin [26,27]. Toxins produced by gut microbiota may weaken the bond between tight junction proteins in the intestinal epithelium, allowing bacteria and their components to enter the bloodstream and trigger inflammatory responses [28]. An increase in lipids induces the desialylation of chylomicrons, i.e., lipoprotein particles produced in the intestine to facilitate the transport of triglycerides and cholesterol [27]. Chylomicrons interact with lipoprotein lipase (LPL) at capillary walls, releasing fatty acids for uptake by cells and accumulation in the intima, contributing to plaque formation. Desialylation of low-density lipoprotein (LDL) increases the uptake by macrophages [29]. Lipid-rich macrophages (foam cells) accumulate in plaque [27]. Lipopolysaccharides (LPS) contribute to the formation of foam cells [28].

Butyrate, produced in the colon by members of the genera Bifidobacterium, Lactobacillus, Enterococcus, Lachnospiraceae, Ruthenibacterium, Flavonifractor, Blautia (previously Ruminococcus), Faecalibacterium, Roseburia, Eubacterium, Anaerostipes, Coprococcus, Subdoligranulum, Anaerobutyricum, and Oscillospira [30,31,32,33,34,35,36,37,38], crosses the gut wall, enters the circulatory system, and is transported via the portal vein to various organs [39], where it is converted to glucose via gluconeogenesis [40,41]. Butyrate prevents the infiltration of macrophages into VSMCs by suppressing the interaction between epidermal growth factor (EGF) and its receptor (EGFR) [42]. This decreases the production of pro-inflammatory cytokines and suppresses plaque formation [43]. Although the inactivation of EGFRs is a promising approach to prevent or repress AS, the accumulation of unbound EGF may induce cell cycle arrest, suppress VSMC growth, and possibly nullify the maintenance role fulfilled by Notch3 [44]. Wang et al. [44] however, suggested that a novel Notch3-mediated signaling pathway may be involved in the maintenance of VSMCs, or that Notch3 may be cross-regulated by other signaling pathways. Indeed, a more recent study [45], showed that a small population of VSMCs produces Notch2. The role of Notch2 in AS is, however, unknown. For a detailed discussion on Notch signaling pathways, the reader is referred to Zhou et al. [46].

Binding of butyrate to peroxisome proliferator-activated receptor γ (PPARγ) increases the activity of IkBα, an inhibitor of NF-κB [47]. This, and the ligation of PPARγ with p65, degrades the NF κB/p65 complex [48], resulting in downregulation of the NF-κB pathway [49,50], the suppression of pro-inflammatory cytokines, and an increase in anti-inflammatory cytokines [51,52]. Butyrate may also exert anti-inflammatory activities by inhibiting interferon γ (IFN-γ) signaling. This suppresses the activity of CD36 and decreases the uptake of ox-LDL by macrophages [51,52,53,54]. Furthermore, butyrate suppresses the NOD-, LRR-, and pyrin domain-containing protein 3 (NLRP3) inflammasome, which alleviates inflammation and scar tissue formation [55,56].

Several arteries have been evaluated as conduits in coronary artery bypass graft (CABG) surgery. Despite experiments with radial, gastroepiploic, inferior epigastric, splenic, subscapular, inferior mesenteric, descending branch of the lateral femoral circumflex, and ulnar arteries, IMAs remain the “golden standard” as CABG. For a detailed discussion on the topic, the reader is referred to He [57].

The first reported use of IMAs for coronary artery bypass grafting (CABG) in humans was in 1961 [58]. Five years later, Favaloro [59] introduced bilateral IMA implants, which instigated the use of IMAs in coronary artery bypass surgery. For further information on IMAs as CABGs, the reader is referred to the review by Squiers and Mack [60]. A testimony to the success of IMA grafting is patency rates of above 90% reported over a 5-year period [61,62,63]. Patency rates of 100% have been reported for pediatric patients 5 to 13 years after surgery [64].

Clinical implications using IMAs as CABGs compared to SVGs include improved long-term survival, reduced risk of myocardial infarction and angina, and a lower risk of repeat revascularization [65]. Direct comparisons between IMAs and other CABGs in humans are lacking. Most of these studies were performed on animals. In one case, a coronary artery taken from an explanted heart with coronary artery disease was used, but without success [57].

This review compares the anatomical and physiological differences between coronary arteries and IMAs. The key physiological differences between coronary arteries and IMAs are listed in Table 1, and the pathological differences between these arteries are listed in Table 2. Compared to the main heart arteries, IMAs are less prone to inflammation and AS. The question is whether this phenomenon is ascribed to anatomical and physiological differences between these arteries, or do IMAs react differently to signals generated by gut microbiota? As explained in this review, signals generated by gut microbiota play a major role in the development of coronary AS. Key microbial reactions are highlighted.

## 2. Location of Coronary Arteries and Internal Mammary Arteries (IMAs)

Coronary arteries branch from the proximal (root) of the aorta and run along the coronary sulcus of the heart muscle (myocardium), located in the middle layer of the heart wall (Figure 1, left image). The right coronary artery (RCA) emerges from the anterior ascending aorta, feeding blood to the right ventricle, the right atrium, and the sinoatrial (SA) and atrioventricular (AV) nodes, which regulate heart rhythm. The RCA divides into the right posterior descending artery (PDA) and the acute marginal artery. The PDA supplies blood to the posterior one-third of the interventricular septum. The left main coronary artery (LMCA) is separated into two branches, the left anterior descending artery (LAD) that supplies blood to the anterior part of the left ventricle and two-thirds of the interventricular septum (Figure 1, left image). The left circumflex artery (LCx) supplies blood to the left atrium and the posterior-lateral part of the left ventricle. The obtuse marginal artery (OMA), diagonals, and septal perforator (SP) are small branches of the coronary arteries. Stenosis of the LAD affects the anterior septum, anterior free base, apical segments of the septum, and anterior wall. Stenosis of the LCx affects the anterolateral wall and the inferolateral wall. Stenosis of the PDA affects the inferior septum and inferior free wall.

The right and left IMAs stem from the proximal area of the brachiocephalic trunk and subclavian artery, respectively (Figure 1, right image), and are positioned under the fascia and deep in the intercostal muscles [66]. In the proximal part, IMAs separate into vessels that supply the breast, thymus, mediastinum, and sternum (Figure 1, right image). The brachiocephalic artery, also known as the innominate artery, splits into two arteries (the right common carotid artery and the right subclavian artery). On the right and left side of the sternum, the subclavian artery gives rise to the vertebral artery, internal thoracic artery, thyrocervical trunk, costocervical trunk, and dorsal scapular artery before diverging into the axillary artery when it crosses the lateral border of the first rib (Figure 1, right image). The two IMAs travel along the inner surface of the anterior chest wall on both sides of the sternum and divide into two branches (musculophrenic and the superior epigastric arteries) at the sixth or seventh intercostal cartilage [67] (Figure 1, right image). The superior epigastric artery flows along the abdominal wall and connects with the inferior epigastric artery. The musculophrenic artery supplies the diaphragm with blood. In the proximal part, the internal thoracic artery divides into vessels that supply the breast, thymus, mediastinum, and sternum (Figure 1, right image). Anterior and posterior branches are formed at each intercostal rib (Figure 1, right image). The luminal diameter of the left and right IMAs decreases from proximal to distal, with 2.6 ± 0.4 to 2.4 ± 0.4 mm, respectively, between the second and fifth rib [81].

## 3. Anatomy of Coronary Arteries and Internal Mammary Arteries

**Figure 2:**
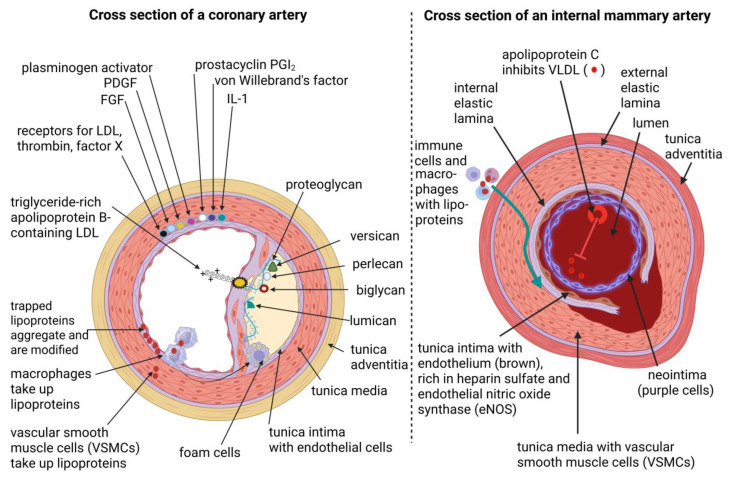
Cross-sections of a coronary artery (**left image**) and an internal mammary artery (IMA, **right image**). The yellow area in the coronary artery denotes plaque. Coronary arteries and IMAs have the same concentric layers, although they differ on a cellular level, as explained in the text. The inner layer (tunica intima or intima) is lined with smooth, longitudinal endothelial cells, a subendothelial layer with connective tissue, and smooth muscle cells. The thick middle layer (tunica media or media, pink layer in both images) is composed of multiple layers of smooth muscle cells connected by elastic fibers, collagen, and proteoglycans. The external layer (tunica adventitia or adventitia), depicted in yellow in the left image and dark pink in the right image, is composed of connective tissue, specifically collagen and elastic fibers. The tunica intima of IMAs (**right image**) contains endothelial cells and neointima. PDGF = platelet-derived growth factor, FGF = fibroblast growth factor, LDL = low-density lipoprotein, IL-1 = interleukin-1, VLDL = very-low-density lipoprotein. The plus symbols (**left image**) denote a positively charged triglyceride-rich apolipoprotein. The red circles in the lumen (**right image**) denote very low-density lipoproteins (VLDLs). Created using Biorender.com (accessed on 4 September 2025).

A cross-section of a coronary artery (with AS; yellow area) is shown in Figure 2 (left image). The histology of the coronary arterial wall is similar to all arteries and is composed of three concentric layers, i.e., an inner (luminal) layer, the intima (tunica intima); a middle layer, the media (tunica media); and an outer (external) layer, the adventitia (tunica adventitia). The tunica intima is lined with smooth, longitudinally orientated endothelial cells (Figure 2, left image), connective tissue, an internal elastic lamina, and is permeable, allowing the diffusion of particles, but serves as a firm barrier between the blood/lumen and the other wall layers. Vascular smooth muscle cells are primarily found in the tunica media, the middle layer of the vessel wall. Endothelial cells in the tunica intima of coronary arteries contain prostacyclin (PGI_2_), von Willebrand’s factor, interleukin-1 (IL-1), plasminogen activator, platelet-derived growth factor (PDGF), and fibroblast growth factor (FGF) (Figure 2, left image). The endothelium spanning the plaque (atheroma, indicated in yellow) is attached to proteoglycans that are linked to negatively charged biglycan, versican, perlecan, and lumican (Figure 2, left image). On a microscopic level, IMAs (Figure 2, right image) share features with elastic-type arteries, e.g., the aorta, and muscular vessels (e.g., the left anterior descending coronary artery) [82]. The tunica intima of IMAs is composed of endothelial cells and neointima (a layer of smooth muscle cells; Figure 2, right image) [68]. Neointima, while a component of the tunica intima, is not always present and is more accurately described as a thickening of the tunica intima in response to injury or disease. The tunica intima is separated from the tunica media by an internal elastic membrane (internal elastic lamina, elastica interna; Figure 2, right image). The tunica media is composed of multiple layers of smooth muscle cells connected by elastic fibers, collagen, and proteoglycans (Figure 2, right image). The cells are embedded in a glycoprotein matrix and are separated from the tunica adventitia by an external elastic membrane (external elastic lamina, elastica externa), composed of elastin (Figure 2, right image). The surface of the external elastic membrane is covered with non-myelinated nerve axons. The tunica adventitia consists of longitudinally orientated collagen and elastic fibers, surrounded by vasa vasorum (small blood vessels that supply and drain arterial walls), nerves, and lymphatic vessels. The endothelium of IMAs has fewer pores and is thus less permeable compared to leg blood vessels (saphenous vein grafts, SVGs), also used in CABG [71]. The tunica media of IMAs (Figure 2, right image) contain circumferentially aligned VSMCs surrounded by longitudinal and circular interlacing elastic fibers [63,64]. The non-fragmented internal elastic lamina with coarse folds prevents the migration of VSMCs [71].

## 4. Physiological Differences Between Coronary and Internal Mammary Arteries

The surface of endothelial cells in the tunica intima of healthy coronary arteries is covered with receptors for LDL, thrombin, and factor X (Figure 2, left image). The endothelium of healthy coronary arteries produce prostacyclin PGI_2_ (an antithrombotic agent), von Willebrand’s factor (a prothrombotic agent), interleukin-1 (IL-1), plasminogen activator (a fibrinolytic agent) that maintains blood flow and dissolves blood clots, platelet-derived growth factor (PDGF) that is released from platelets and repair blood vessels, and fibroblast growth factor (FGF) that drives tissue repair and metabolism (Figure 2, left image). IL-1 induces endothelial cells to recruit infiltrating leukocytes and trigger the expression of the protein tissue factor (TF) that acts as a procoagulant.

In atherosclerotic arteries, the tunica intima spanning the plaque structure attracts proteoglycans associated with positively charged lipoproteins, specifically apolipoprotein B-containing LDL particles [69] (Figure 2, left image). Lumican, a proteoglycan (Figure 2, left image), is actively expressed by the tunica intima of atherosclerotic carotid arteries [72]. Lipoproteins are taken up by macrophages and form lipid-rich macrophages (foam cells), which are deposited in the lumen of coronary arteries, initiating the formation of plaques (Figure 2, left image). Negatively charged side chains of proteoglycans, such as biglycan, versican, perlecan, and lumican, are over-expressed in the tunica intima of atherosclerotic carotid arteries (Figure 2, left image) [72]. The proteoglycans interact with positively charged triglyceride-rich apolipoprotein B-containing LDL particles (Figure 2, left image) [69]. Plaque formation and AS may thus be controlled by preventing the accumulation of lipoproteins, specifically apolipoprotein B-containing LDL particles, proteoglycans such as lumican, biglycan, versican, perlecan, and lumican. The accumulation of lipoproteins may be prevented by destroying or blocking lipid-binding receptors on the tunica intima, or by preventing the exchange of lipids between lipoproteins by suppressing the lipid transfer protein (LTP). Another option would be to suppress the synthesis of apolipoprotein B on a mRNA level. Inhibiting the synthesis of proteoglycans is possible by using compounds that interfere with glycosaminoglycan chain reactions. The target of such compounds must be specific. If not, the synthesis of all proteoglycans may be inhibited, which may negatively impact important biological processes such as the repression of tumors and the healing of scar tissue.

The tunica intima of IMAs (Figure 2, right image) is not more elastic than the tunica intima of coronary arteries. The elastic fibers of IMAs are embedded within VSMCs and collagen. Furthermore, the tunica intima of IMAs is not invaded by VSMCs, which contributes to its elasticity and sustained thickness [73,76]. eNOS is actively expressed in the endothelium of IMAs, which increases NO levels and dilates the arteries. NO inhibits the expression of monocyte chemoattractant protein-1 (MCP-1), thus preventing the recruitment of monocytes [73]. NO also increases the expression of prostaglandin, which relaxes VSMCs (reduces blood pressure), and prevents plaque formation [75,83]. An increase in NO coincides with a decline in GTP cyclohydrolase (GTPCH) and tetrahydrobiopterin (BH4 or THB) [74]. A decline in GTPCH lessens hypertension and reduces plaque formation [80]. The opposite is also true. An increase in GTPCH and BH4 activity induces the uncoupling of eNOS, leading to the production of cell-damaging ROS, and the commencement of AS [84,85,86,87]. Further research on the inhibition of GTPCH and the role of BH4 in other biological processes may lead to the developing of targeted therapies.

Endothelial cells of IMAs produce bradykinin, which stimulates the release of NO. The level at which bradykinin regulates the release of NO in IMAs is, however, much lower than observed for gastroepiploic and inferior epigastric arteries [88]. This suggests the functioning of bradykinin may be specific to the type of vessel. From a pain-regulation level, this would make sense, as bradykinin is a potent pain-producing molecule and interacts with prostaglandins to increase pain sensation. Prostaglandins also regulate inflammation, blood clotting, and reproductive processes [89]. Further research is required to understand the role bradykinin plays in regulating IMA function, especially since IMAs are commonly used in CABG. The activity of bradykinin is influenced by other vasoactive substances and pathways, such as the renin-angiotensin system and angiotensin-converting enzyme (ACE) inhibitors [90].

The endothelium of IMAs has fewer fenestrations compared to coronary arteries, is less permeable, and tissue-type plasminogen activator (tPA) produced by VSMCs helps prevent clot formation [76]. The tunica intima of IMAs has fewer pores compared to the tunica intima of coronary arteries and leg veins (saphenous veins) that are used in CABG [68]. This could prevent lipoproteins from entering the subendothelial space. Macrophages accumulate at the medial/adventitial border of IMAs [91], suggesting that immune cells migrate along the outer surface of the endothelium (shown as a blue arrow in Figure 2, right image), potentially contributing to vessel wall maintenance and immune responses within the artery. The low-density vasa vasorum (small blood vessels) in IMAs limit the trafficking of immune cells, promoting scar formation in the tunica intima and reducing plaque buildup [79]. It thus seems as if the unique composition of the IMA extracellular matrix is key to the absence of cholesterol build-up (plaque formation) and AS. Indeed, Shao et al. [92] and Vij et al. [93] have shown that the difference in composition of the extracellular matrix of the tunica intima of IMA compared to the extracellular matrix of AS-susceptible arteries is key in preventing the accumulation of plaque-forming lipids and a decrease in the migration of immune cells.

Enzymatic studies conducted on IMAs removed from patients with acute coronary syndrome or chronic stable angina have shown low expression of inducible nitric oxide synthase (iNOS) and intercellular adhesion molecule-1 (ICAM-1) [77]. Blood flow in IMAs is regulated by the production of prostaglandin I2, endothelin-1, tissue plasminogen activator, ICAM-1, and transforming growth factor-β1 (TGF-β1) [68]. It is hypothesized that the abundant collateral blood supply of IMAs may offer some protection against AS [94]. This requires further research. Furthermore, the inner diameter of IMAs is similar to that of coronary arteries, which allows less turbulent flow compared to saphenous vein grafts (SVGs) with larger inner diameters [68].

The uptake of LDL by endothelial cells in carotid arteries is driven by a scavenger receptor class B type 1 (SR-B1) protein [95], thus promoting atherogenesis. The transition of VSMCs to macrophage-like cells may rupture plaques. On the other hand, VSMC-derived fibroblast-like cells (fibromyocytes) may strengthen the fibrous cap (area facing the vascular lumen) of the plague [96]. Changes in the level of VSMCs are regulated by pulsatile (rhythmic) stress, growth factors such as platelet-derived growth factor (PDGF) BB secreted by endothelial cells [97], thrombin [98], and monocytes [99]. The VSMCs of IMAs are resistant to these stimuli, despite the presence of PDGF and thrombin receptors [100]. The reason for PDGF receptors in IMAs may be associated with the triggering of MAPK, which upregulates the cyclin-dependent kinase inhibitor p27Kip1 required to activate VSMCs [99,101].

Overexpression of the *APOC3* gene, encoding the apolipoprotein C-III (apo-CIII), inhibits the buildup of very-low-density lipoprotein (VLDL) (Figure 2, right image) and represses the development of AS [102]. IMAs are thus less prone to AS and remain stable over a longer period [103]. Sims [70] has shown that the width and stiffness of the tunica intima in IMAs remained unchanged in individuals older than 50 years, as opposed to the tunica intima of coronary arteries that increased in thickness in patients older than 30 years. Ruengsakulrach et al. [103] have shown that, in contrast to IMAs, radial arteries (RAs) that stem from brachial arteries are more likely to develop AS, accumulate cells in the vascular intima, and calcify the medial region. Furthermore, fewer atheromatous lesions were observed in IMAs compared to RAs. [103].

**Figure 3:**
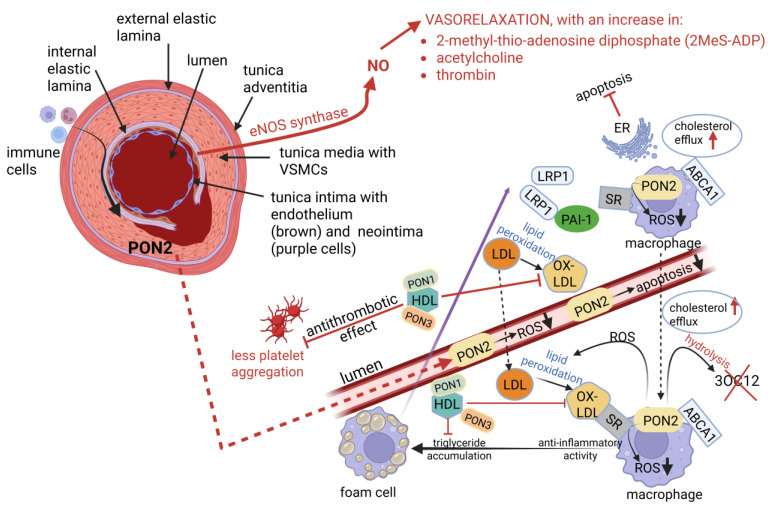
The role of paraoxonase (PON) in IMAs. PON2 is highly expressed in the VSMCs of IMAs but not in atherosclerotic arteries. PON2 lowers the production of reactive oxygen species (ROS), decreases apoptosis, and protects mitochondria against oxidative stress, and the endoplasmic reticulum (ER) against stress-induced apoptosis (**right image**). PON1 regulates reverse cholesterol transport and is antioxidative, anti-inflammatory, antiapoptotic, vasodilative, and antithrombotic. PON2 and PON3 are attached to high-density lipoprotein (HDL) and oxidize lipid peroxides to prevent their accumulation on low-density lipoprotein (LDL). Oxidized LDL (ox-LDL) converts macrophages to foam cells. Foam cells and the release of pro-inflammatory cytokines from adipose tissue led to plaque formation. Platelet aggregation is prevented by the HDL-PON complex. ATP-binding cassette transporter A1 (ABCA1) mediates the cellular efflux of phospholipids and cholesterol to lipid-poor apolipoprotein A1 (apoA1)-HDL. Lighter circles in the foam cell represent the accumulation of triglycerides (lipids). LRP1 = low-density lipoprotein receptor-related protein 1, PAI1 = plasminogen activator inhibitor 1, ABCA1 = ATP-binding cassette transporter A1, SR = scavenger receptor. Red arrows pointing upwards denotes an increase, and black arrows pointing downwards a decrease. Red blunt-ended arrows denote inhibition. The red cross denotes no formation of the molecule. Created using Biorender.com (accessed on 4 September 2025).

Paraoxonase 2 (PON2) is highly expressed in VSMCs of IMAs (Figure 3) but not in carotid arteries [70]. The antiatherogenic effect of PON2 (Figure 3) is attributed to the lowering of reactive oxygen species (ROS) and apoptosis, reviewed by Dicks [21]. PON2 also protects mitochondria against oxidative stress [104] and the endoplasmic reticulum (ER) against stress-induced apoptosis [105] (Figure 3). PON1 (Figure 3) regulates reverse cholesterol transport and is antioxidative, anti-inflammatory, antiapoptotic, vasodilative, and antithrombotic [78]. PON2 (and PON3) has a strong lactonase activity which hydrolyzes the bacterial signaling molecule N-3-oxododecanoyl homoserine lactone (3OC12) [106] (Figure 3). 3OC12 also interacts with the human immune defense system, especially polymorphonuclear neutrophils (PMNs) that are the first to enter an infected site [107]. The activity of PON1 is stimulated by its association with HDL, high concentrations of certain salts, and potentially by other factors like antioxidants and specific drug treatments. We need to understand how these factors modulate PON1 activity to mitigate oxidative stress and AS.

## 5. The Role of Gut Microbiota in Atherosclerosis (AS)

**Figure 4:**
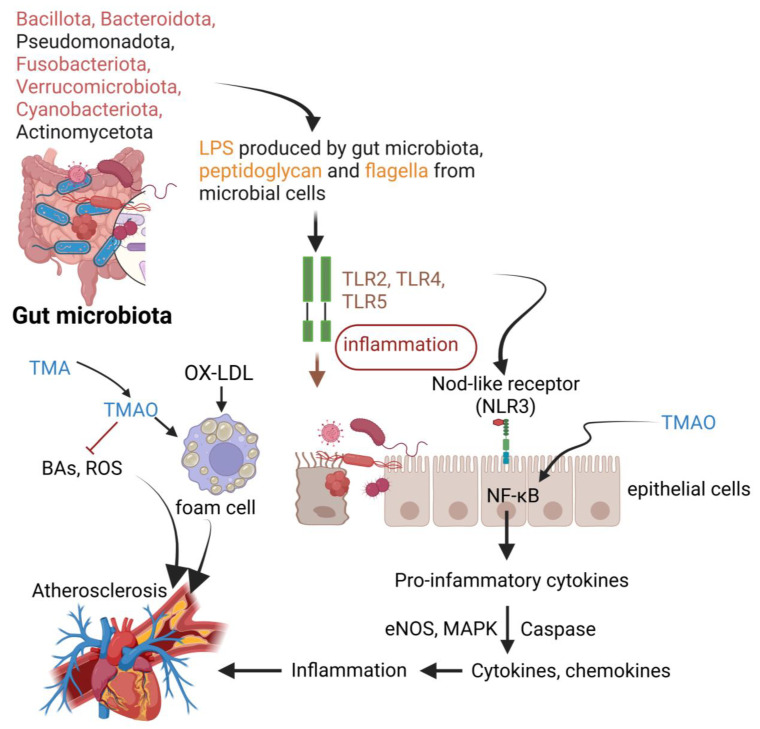
The influence of gut microbiota on the formation of atherosclerosis (AS). Lipopolysaccharides, peptidoglycan, and flagella proteins, produced by gut microbiota, enter the bloodstream and react with specific Toll-like receptors, leading to systemic inflammation and disruption of intestinal epithelial cells. Toll-like receptors react with Nod-like receptors such as NLR3, stimulating nuclear factor kappa-B signaling and mitogen-activated protein kinase pathways, fueling inflammation. The inflammatory responses, microbial conversion of trimethylamine to trimethylamine N-oxide, and the accumulation of oxidized low-density lipoprotein in foam cells (high-lipid containing macrophages) lead to plaque formation. Trimethylamine N-oxide represses the production of bile acids and reactive oxygen species. LPS = lipopolysaccharides, TMA = trimethylamine, TMAO = trimethylamine N-oxide, BAs = bile acids, ROS = reactive oxygen species, OX-LDL = oxidized low-density lipoprotein, TLR = Toll-like receptor, NF-κB = nuclear factor kappa beta, eNOS = endothelial nitric oxide synthase, MAPK = mitogen-activated protein kinase. Created using Biorender.com (accessed on 30 June 2025).

The impact of gut microbiota on AS is summarized in Table 3. The gut microbiome of healthy individuals is composed of Bacillota (previously Firmicutes), Bacteroidota (previously Bacteroidetes), Pseudomonadota (previously Proteobacteria), Fusobacteriota (previously Fusobacteria), Verrucomicrobiota (previously Verrucomicrobia), Cyanobacteriota (previously Cyanobacteria), and Actinomycetota (previously Actinobacteria) (Figure 4). Individuals diagnosed with AS and other CVDs have less Proteobacteria, and Actinobacteria [104] and have a higher tendency to produce lipopolysaccharides [108] (LPS, Figure 4). The binding of microbial LPS and other microbial cell components to TLRs, especially TLR4, bacterial flagella to TLR5, and peptidoglycan to TLR2, induces inflammation and immune responses, leading to drastic changes in intestinal permeability [109,110] (Figure 4). An increase in the expression of NLRs, especially NLRP3, activates NF-κB and MAPK pathways, which in turn lead to an increase in the expression of cytokines and chemokines (Figure 4). A constant increase in inflammatory responses may lead to AS [111,112] (Figure 4). Activation of the TLR4 pathway leads to an increase in eNOS and MAPK/NF-κB pathways (Figure 4). Oxidative stress leads to the formation of ox-LDL, which is a potent inducer of macrophage foam cells (Figure 4). T cell (Treg) differentiation and prostaglandin production are regulated by specific microbial components that induce the production of type I interferon (IFN) in macrophages and dendritic cells [113]. The regulation of Treg differentiation and prostaglandin production by gut microbiota is important in maintaining intestinal immune homeostasis and preventing inflammatory diseases. Enterocytes and mucus-producing goblet cells play important roles in immune responses by interacting with gut microbiota and the host’s immune system. Immunoglobulin A (IgA), secreted by goblet cells and present in intestinal mucus, plays a critical role in regulating the composition and activity of the gut microbiota, influencing overall gut and systemic health [114].

Bifidobacterium lactis [133], Lactiplantibacillus plantarum (previously Lactobacillus plantarum) [134], and pathogenic bacteria such as Salmonella enterica (previously Salmonella choleraesuis), Listeria monocytogenes, and Helicobacter pylori (previously Campylobacter pylori) [135] capture human plasminogen on their cell surface and convert it to plasmin, a protease that can degrade extracellular matrix components and fibrinogen [131]. This interaction can influence bacterial colonization, inflammation, and potentially contribute to AS. Elevated levels of plasminogen activator inhibitor type-1 (PAI-1) are linked to increased risk of AS, thrombosis, and other CVDs due to its ability to regulate the fibrinolytic system and inhibit the breakdown of blood clots [132]. By increasing the expression of PAI-1 in IMAs, fibrinolysis may be attenuated and plaque formation repressed. However, several studies indicated that PAI-1 may be a risk factor for CVD, including MI, stroke [136,137,138], coronary heart disease [139], venous thrombosis [140], and AS [141]. These findings need to be verified by considering other risk factors, e.g., metabolic abnormalities, age, and sex.

SCFAs produced by gut microbiota increase the production of insulin-like growth factor 1 (IGF-1), which affects the vascular tone of IMAs and the development of the tunica intima. This may lead to graft failure [115]. In obese individuals, the accumulation of fat (lipogenesis) and, often, resistance to insulin causes mitochondrial dysfunction, systemic or local inflammation [142,143], dysbiosis, and the activation of signaling pathways that increase inflammation and oxidative stress [144]. An imbalanced gut microbial population leads to a decrease in the production of short-chain fatty acids (SCFAs), including butyrate. SCFAs are important in the regulation of histone deacetylase (HDAC), thus gene expression, reviewed by Dicks [21]. A decrease in SCFAs may also lead to a decrease in activated GPR41 and an accumulation of carbohydrates [116]. SCFAs produced in the GIT play an essential role in promoting the secretion of glucagon-like peptide 1 (GLP-1) and peptide YY (PYY) [145]. GLP-1 increases the secretion of insulin [146], and PYY reduces food intake, inhibits intestinal motility, and stimulates bowel movement [116]. Butyrate and propionate inhibit tumor necrosis factor (TNF) and NF-κB signaling pathways [117], and stimulate NLRs [118] to modulate inflammatory responses [119]. Butyrate, propionate, and acetate binds to GPR41, GPR43, and GPR109a, leading to the activation of MAPK/extracellular signal-regulated kinase (ERK) and p38MAPK signaling pathways. The binding of SCFAs with GPR41 promotes the production of interleukin (IL)-22 in CD4+ T cells [120]. For more information on the role of butyrate in anti-inflammatory responses, the reader is referred to the review by Dicks [147]. Butyrate also lowers diastolic blood pressure (DPB) [121] and reduces the risk of developing AS [122].

The anti-inflammatory properties of butyrate are well known and are addressed elsewhere in this review. Butyrate down-regulates genes involved in lipid metabolism, including acyl-CoA thioesterase1 (Acot1), Acot2, Perilipin2 (Plin2), Plin5, and Cytochrome4a (10, 14, and 31 isoforms) [123]. Butyrate also activates ATP-binding cassette subfamily A member 1 (ABCA1), which reduces total cholesterol (TC) levels and suppresses plaque formation [123]. Furthermore, butyrate downregulates the overproduction of vascular cell adhesion molecule 1 (VCAM-1) and E-selectin, which prevents the adhesion of monocytes to injured endothelia [124].

As described elsewhere in this review, binding of butyrate to PPARγ increases the activity of IkBα, resulting in downregulation of the NF-κB pathway, the suppression of pro-inflammatory cytokines, and an increase in anti-inflammatory cytokines. The increase in eNOS and NO production is regulated by proteins p22phox and p47phox, which are key components of NADPH oxidase (NOX) [148]. Further research is required to determine if the production of p22phox and p47phox is upregulated in IMAs. This may explain why ROS levels are higher in IMAs. For further information on how IMAs react to immunological changes, compared to coronary arteries, the reader is referred to the review by Dicks [147].

Most studies support the positive effect of butyrate on CVDs. However, a recent study [149] has shown that 3.9 g of sodium butyrate, orally administered to 23 patients daily for 4 weeks, increased their daytime systolic and diastolic blood pressure. This may cause damage to restricted arteries. These findings could, however, not be confirmed with studies on mice [149]. Further research is required to understand the interaction between butyrate and its receptors pertaining to the regulation of blood pressure. A study conducted by Gee et al. [150] has shown that the activation of NF-κB may accelerate certain cardiovascular abnormalities. The hypothesis is that NF-κB induces the transformation of endothelial cells into mesenchymal cells in the aorta, which leads to calcification and stenosis, characteristic of calcific aortic valve disease (CAVD). Although the study did not link NF-κB activation to butyrate, the induction of pathways involved in the production of pro-inflammatory cytokines (e.g., TNF-α) instigated an endothelial-to-mesenchymal transformation in aortic valve endothelial cells. For further information on the preventative role of butyrate in AS and cardiovascular health, the reader is referred to the review by Dicks [130].

Bile acids (BAs), produced from microbial metabolism, prevent AS by regulating vascular tension and altering ion exchange through cardiomyocyte membranes [125]. However, the regulation of AS may rely on many other interactions, such as preference for binding to Farnesoid receptors (FXRs) or GPRs. Binding of BAs to these receptors reduces atherosclerotic plaque formation and inhibits phagocytosis of ox-LDL by macrophages [126,127]. More information is available in the review by Dicks [21]. Gut microbiota and their metabolites (especially secondary BAs) play crucial roles in hypertension, AS, unstable angina, and heart failure.

Trimethylamine N-oxide (TMAO), produced by gut microbiota, increases the release of intracellular calcium ions, activates platelet formation, increases the risk of developing AS (Figure 4), and promotes thrombosis [128]. AS is promoted due to the interaction of TMAO on the sirtuin 3-superoxide dismutase 2-mitochondrial ROS pathway, the ROS-thioredoxin interactive protein axis [151], and the protein kinase C/NF-κB (canonical NF-κB)/vascular cell adhesion molecule-1 pathway [129]. TMAO activates NLRP3 and triggers platelet formation [130]. Information on NLRP3 production and TMAO in IMAs is limited and warrants further investigation to understand why IMAs are less prone to platelet formation.

## 6. Aging Arteries and Changes in the Gut Microbiome Accelerates Atherosclerosis (AS)

The aging of arteries is synonymous with damage to endothelial membranes and the development of AS, reviewed by Dicks [152]. Aging is associated with an increased release of autocrine and paracrine signals, causing physiological dysfunction. The recruitment of immune cells to inflamed arteries, including coronary arteries, and an increase in the uptake of ox-LDL by macrophages (foam cells) restricts blood flow. The inability of aging and damaged ECs to accommodate vast changes in signaling molecules, many produced by gut microbiota, leads to a range of anatomical and physiological arterial anomalies, such as the degradation of cardiovascular membranes, fibrosis, calcification, plaque formation, and a dysfunctional immune system, reviewed by Dicks [152].

Aging arteries release senescence-associated secretory phenotype (SASP) factors, e.g., matrix metalloproteinase 9 (MMP-9). These upregulate the inflammasome and stimulate healthy endothelial cells and VSMCs to produce pro-inflammatory cytokines [153,154,155]. Metalloproteinases initiate the lysis of collagen. This, together with the degradation of elastin, destabilizes the fibrous cap of plaques, increasing the risk of rupture and thrombosis [153,155]. Elderly individuals with a weakened immune system are unable to eliminate dead cells, resulting in the further release of SASP [156]. The infiltration of vessel walls by senescent immune cells increases the production of pro-inflammatory cytokines and chemokines [156]. Furthermore, TET methylcytosine dioxygenase 2 (TET2)-deficient macrophages, abundant in aging blood cells, increase the release of NLRP3 inflammasome-mediated interleukin-1β (IL-1β) [153]. These physiological changes lead to the formation of larger aortic plaques [157]. The loss of interstitial cells of Cajal [158] by an aging gut suppresses cholinergic motor nerve functions [159]. This, together with a decline in enteric glial cells [160], an increase in senescence-like activity within myenteric nerve cell bodies [161], and an increase in total collagen content within muscles and the submucosal plexus [162], accelerates AS.

As the immune system ages, the hematopoietic stem cells (HSCs) become impaired, which leads to myelopoiesis and a decline in lymphoid cells [163]. With aging, fewer naïve T and B cells are produced from bone marrow, resulting in a decline in the production of lymphoid progenitor cells [164]. Drastic changes in the composition of gut microbiota alter the expression of TLRs and NLRs by DCs in direct contact with gut microbiota and naïve T-cells behave as effector T-cells (TH1, TH2, and TH17) or Treg [165]. Tregs produce IL-10 and TGF-β, which inhibit pro-atherogenic effector T cells and stimulate efferocytosis by macrophages [166]. In aging and atherosclerotic endothelial membranes, the negatively charged biglycan, versican, perlecan, and lumican bind to positively charged lipoproteins, e.g., apolipoprotein B attached to ox-LDL [167]. For further information on aging-related processes and an aging gut microbiome on AS, the reader is referred to the review by Dicks [152].

## 7. Conclusions

The VSMCs of IMAs are more elastic and differ from rigid arteries such as the aorta and the internal carotid. VSMCs are maintained by the Notch 3 protein, which also plays a pivotal role in the regulation of gene transcriptions, intracellular communication, neural development, and the binding of EGF to EGFRs on the surface of VSMCs. Inhibition of EGFRs decreases inflammation and oxidative stress, resulting in a reduction in AS. Arteries protected from AS have less infiltration of macrophages and lower levels of pro-inflammatory cytokines. Arteries prone to AS are penetrated by lipid-containing macrophages (foam cells) and are more prone to fibrosis and the accumulation of ROS. Since EGFRs are activated by toll-like receptors such as TLR4, their disruption may decrease inflammatory activities and repress the production of macrophage foam cells. Although the inactivation of EGFRs and TLR4 sounds like a promising approach to treating AS, the inhibition of EGFR may repress VSMC growth by repressing Notch3. If, however, the Notch3-mediated signaling pathway is regulated by other signaling pathways, the physiology of VSMCs may not be negatively affected. Further research is required to determine the role of Notch2 in maintaining VSMCs. Treatments that support the expression of eNOS (increase in NO release) may prevent AS. AS may be treated by repressing the growth of gut microbiota that produces TMAO. By repressing the production of TMAO, the synthesis BAs is stimulated, which downregulates the expression of membrane glycoprotein CD36 and platelet formation. The treatment of AS requires a better understanding of trimethylamine, N-oxide, short-chain fatty acids, and primary and secondary BA metabolism in the GIT. Extensive metabolomic studies on commensal gut microbiota may open an exciting avenue of research into novel anti-atherosclerotic strategies.

As we age, our gut microbiome changes. This may lead to dysbiosis and the production of excessive succinate, fumarate, SCFAs, LPS, and toxins that alter the permeability of the intestinal epithelium and increase cytokine levels. Further research is required to understand the effect of pro-inflammatory cytokines on endothelial cells in the tunica intima of IMAs and coronary arteries. In-depth studies on the mechanical resistance of IMAs may aid in understanding the stability of tight junctions in endothelial cells, elasticity, conditions that support an increase in NO production, and factors that suppress selectins and other adhesion molecules. Biochemical and physiological reactions rendering IMAs more resistant to atherogenesis need to be studied using multi-omics and systems biology approaches. We need to have a better understanding of how molecules and structures of IMAs interact with each other. Metagenomic, metabolomic, and proteomic studies may shed light on how a changing gut microbiome alters our immune responses as we age. In-depth studies on immunosenescence and dysfunctional endothelial cells may provide more answers to the prevention of AS and CVDs. Novel anti-atherosclerotic treatment strategies may also lead to the discovery of new targets to diagnose AS and CVDs, especially at an early stage before symptoms develop.

## Figures and Tables

**Table 1:** Location, function, and key physiological differences between coronary arteries and internal mammary arteries (IMAs).

Characteristic	Coronary Arteries	Internal Mammary Arteries (IMAs)	References
Location	Branches directly from the ascending aorta and run along the surface of the heart muscle (myocardium), with smaller penetrating branches	Ascend from the left and right proximal subclavian arteries and are located on the inside of the rib cage, one on each side of the sternum.	[57,66]
Main function	Supplies oxygen-rich blood to the myocardium	Supplies blood to the anterior chest wall, including the sternum and breasts.	[57,67]
Blood flow	Rhythmic, with most blood flow during diastole (relaxation phase)	Steady, but blood flow can alter in response to demand.	[57,68]
Atherosclerosis (AS)	Plaque buildup due to the deposit of oxidized low-density lipoproteins (oxLDL), calcium, fibrin, and cellular debris onto the inner lining (tunica intima) of arterial walls, leading to hypertension and coronary artery disease (CAD)	Highly resistant to plaque buildup and AS, due to unique cellular and structural properties, and increased production of apolipoprotein C-III (apo-CIII), which inhibits the buildup of LDL and plaque formation. Paraoxonase 2 (PON2) expressed in VSMCs lowers the production of reactive oxygen species (ROS). PON1 regulates reverse cholesterol transport, is antioxidative, anti-inflammatory, anti-apoptotic, vasodilative, and antithrombotic.	[69,70]
Internal elastic lamina	Fenestrated, and supports the migration of vascular smooth muscle cells (VSMCs), and intimal hyperplasia	Non-fenestrated and forms a barrier preventing intimal hyperplasia and AS.	[71]
Tunica intima	Composed of smooth, longitudinally orientated endothelial cells (ECs), connective tissue, and an internal elastic lamina. Thickness increases with age. Prone to the formation of lesions	Similar in composition, but thinner and lacks a prominent internal elastic lamina. The neointima is separated from the tunica media by an internal non-fragmented (and less defined) internal elastic lamina. Rich in heparin sulfate and endothelial nitric oxide synthase (eNOS). The increased production of nitric oxide (NO) relaxes VSMCs and suppresses GTP cyclohydrolase (GTPCH), thereby lowering blood pressure and reducing plaque formation. Higher levels of prostacyclin (PG12) inhibit platelet aggregation.	[72,73,74]
Arterial blockage	Prone to becoming blocked in CAD	More resistant to blockage and used as grafts in coronary arterial bypass surgery	[57,58]
Clinical significance	Ischemic heart disease, leading to angina and myocardial infarction (MI)	Surgical replacement of blocked coronary arteries with IMAs lowers the risk of developing angina and MI and has superior long-term patency.	[57,58]

**Table 2:** Key pathological differences between coronary arteries and internal mammary arteries (IMAs).

Characteristic	Coronary Arteries	Internal Mammary Arteries (IMAs)	References
Hemodynamic stress	Withstand extreme hemodynamic stress, high pressures, and shear forces.	Less resilient to hemodynamic stress than the coronary arteries.	[75]
Endothelial function	Impaired in atherosclerotic coronary artery disease (ACAD).	Robust endothelial function and are generally more resistant to pathological changes.	[76]
Nitric oxide (NO) production	Basal NO production due to reduced endothelial nitric oxide synthase (eNOS) expression. More susceptible to atherosclerosis (AS) compared to IMAs.	Abundant production of NO, which contributes to its vasodilatory properties and long-term patency.	[72,73,74,77,78]
Vasa vasorum (VV) density	High VV density, but it varies among individual coronary artery branches. The right coronary artery has a significantly higher VV density compared to the left anterior descending coronary branch. The high VV density is crucial for supplying blood to the thickened walls of the coronary arteries.	Lower VV density compared to the coronary arteries. This contributes to the protection of IMAs to AS.	[71,79]
Sympathetic innervation	Dense adrenergic innervation in the media and adventitia, primarily associated with the epicardial arteries and larger vessels.	Sympathetic fibers in the adventitia, though perhaps with lower density. IMAs have less direct sympathetic innervation than the coronary arteries, suggesting they are less subject to neural regulation.	[71]
Exposure to circulating lipids	Directly exposed to high concentrations of circulating lipids, such as cholesterol and triglycerides, making them vulnerable to plaque buildup.	Protected from AS, despite being exposed to the same circulating lipids. This is due to unique biological properties that protect them from plaque buildup.	[69,70,80]

**Table 3:** The impact of gut microbiota on atherosclerosis (AS).

Mechanism	Products Produced by Gut Microbiota and Their Effect on Epithelial and Endothelial Cells	Impact on AS	References
Metabolites produced	Short-chain fatty acids (SCFAs) such as butyrate, acetate, and propionate, reactive oxygen species (ROS), lipopolysaccharides (LPS), trimethylamine-N-oxide (TMAO), and bile acids (BAs)	SCFAs alter the vascular tone of coronary arteries and internal mammary arteries (IMAs), promote the production of inflammatory cytokines, suppress the production of pro-inflammatory cytokines, prevent platelet membrane glycoprotein 4 (CD36) to take up oxidized low-density lipoprotein (ox-LDL), suppress plaque formation, lower diastolic blood pressure (DPB), and prevent the adhesion of monocytes to injured endothelia. SCFAs also increase the production of insulin-like growth factor 1 (IGF-1) ROS leads to the formation of ox-LDL, which is a potent inducer of macrophage foam cells LPS induces inflammation and immune responses, leading to drastic changes in intestinal permeability. TMAO triggers platelet formation, inhibits the synthesis of bile acids (BAs), accelerates the formation of aortic lesions, and upregulates the expression of CD36 on the surface of platelets and epithelial cells. TMAO stimulates the development of macrophage foam cells, increasing the risk of thrombosis. Bile acids affect lipid metabolism and may promote reverse cholesterol transport. Binding of BAs to Farnesoid receptors (FXRs) or G protein receptors (GPRs) reduces the risk of AS.	[15,109,110,113,115,116,117,118,119,120,121,122,123,124,125,126,127,128,129]
Gut barrier integrity	Dysbiosis alters the integrity of the gut barrier by weakening the tight junction proteins.	Increases intestinal permeability, allowing microbial components such as LPS to enter the systemic circulation, which triggers systemic inflammation and AS.	[21,130]
Systemic inflammation	Microbial products activate Toll-like receptor (TLR) signaling in immune cells, promoting the production of inflammatory cytokines.	Systemic inflammation is a key driver of AS, leading to the proliferation of VSMCs, migration of monocytes and T lymphocytes, and the development of lipid-rich macrophages (foam cells). Activation of the Toll-like receptor 4 (TLR4) pathway leads to an increase in endothelial nitric oxide synthase (eNOS) and mitogen-activated protein kinase (MAPK)/nuclear factor kappa B (NF-κB) pathways, which lead to an increase in the expression of cytokines and chemokines.	[27,111,112,113]
Platelet function and thrombosis	Microbial metabolites such as phenylacetylglutamine (PAGln) and plasmin increase the risk of cardiovascular events and heart failure.	PAGln binds to receptors on platelets, increasing the risk of blood clotting. Pathogenic bacteria convert plasminogen to plasmin, a protease that degrades extracellular matrix components and fibrinogen. This stimulates plaque formation, bacterial colonization, and inflammation, contributing to AS.	[131,132]
Immunomodulation	Influences immune responses, particularly via B cell activation through TLR signaling	Activated B cells increase circulating antibodies such as immunoglobulin G (IgG). The differentiation of T-regulatory (Treg) cells and prostaglandin production is regulated by specific microbial components that induce the production of type I interferon (IFN) in macrophages and dendritic cells. The regulation of Treg cells by gut microbiota maintains intestinal immune homeostasis and prevents inflammatory diseases.	[113]
